# Single-stage laparoscopic surgery for bilateral organ tumors using a transumbilical approach with a zigzag incision: a report of two cases

**DOI:** 10.1186/s12894-018-0343-6

**Published:** 2018-05-02

**Authors:** Yoichiro Kato, Renpei Kato, Misato Takayama, Daiki Ikarashi, Mitsutaka Onoda, Tomohiko Matsuura, Mitsugu Kanehira, Ryo Takata, Shigeaki Baba, Toshimoto Kimura, Koki Otsuka, Jun Sugimura, So Omori, Akira Sasaki, Wataru Obara

**Affiliations:** 1 Department of Urology, Iwate Medical University School of Medicine, 19-1 Uchimaru, Morioka, 020-8505 Japan; 2 Department of Surgery, Iwate Medical University School of Medicine, 19-1 Uchimaru, Morioka, 020-8505 Japan

**Keywords:** Reduced port laparoscopic surgery, Transumbilical approach, Zigzag incision

## Abstract

**Background:**

Reduced port laparoscopic surgery (RPLS) is comparable to conventional multiport laparoscopic surgery and has the potential to provide improved cosmesis and decreased pain; as such, it satisfies a growing demand for less invasive surgical procedures. Moreover, a zigzag incision of the umbilicus results in a less visible scar in plastic surgery. Here we report a series of two cases with bilateral organ tumors treated by single-stage RPLS using a combination of a transumbilical approach and a zigzag incision.

**Case presentation:**

Case 1: A 63-year-old man was diagnosed with right renal cell carcinoma (RCC) (clear cell carcinoma, pT1a, venous invasion (−)) and a splenic tumor (cavernous hemangioma). Case 2: An 84-year-old woman was diagnosed with concurrent left RCC (clear cell carcinoma, pT1b, 65 × 65 mm, venous invasion (+)) and ascending colon cancer (adenocarcinoma pT3 with no nodal involvement (0/48)). The perioperative course was uneventful in both cases. However, an additional incision was required in Case 2 for specimen excision. Therefore, the scars were more obvious in Case 2 than in Case 1.

**Conclusions:**

Although more cases are required to evaluate the superiority of this technique, this novel procedure could be considered for patients with bilateral lesions.

## Background

Reduced port laparoscopic surgery (RPLS), especially single-port laparoscopic surgery for adrenalectomy, is recognized as a comparable approach to general laparoscopic surgery in terms of bleeding, complication rate, and operating time [1, 2]. Moreover, aesthetic outcomes and postoperative pain are favorable for RPLS [1, 2]. However, RPLS must be performed with great care and attention because of the difficulty of manipulating forceps in a small space, such as in nephrectomy [3]. A zigzag incision (ZI) has been reported as almost scar-less by plastic surgeons [4]. Hachisuka et al. applied this method with the transumbilical approach and reported being able to maintain the skin’s cosmetic appearance [5]. Therefore, we aimed to perform single-site RPLS for multifocal lesions located at both ends of the body in two patients and followed the each scar.

## Case presentation

The two patients provided informed consent for the publication of their case.

**Fig. 1:**
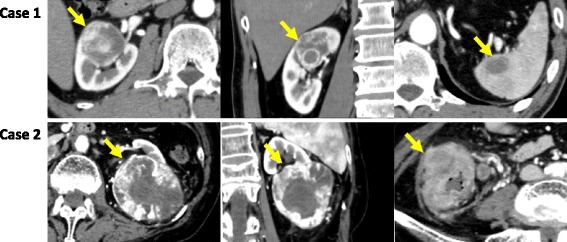
Enhanced computed tomography (CT) showing renal tumor and suspected metastatic tumor or concurrent colon cancer. Above, well-enhanced right renal tumor and splenic tumor of Case 1.Below, large enhanced left renal tumor and ascending colon tumor with lymph node swelling of Case 2

Case 1: A 63-year-old man who was diagnosed with a right renal tumor and a splenic tumor presented to our department. He was asymptomatic, but an ultrasonography scan performed during a routine medical examination revealed a right renal mass. Enhanced computed tomography (CT) showed a 40-mm-diameter right renal mass with enhancement and a 21-mm diameter splenic mass with weak enhancement (Fig. 1). We diagnosed the asses as a right renal cell carcinoma (RCC) and a metastatic splenic tumor (cT1bN0M1) clinically. The patient’s body mass index (BMI) was 22.8 kg/m^2^ (Table 1).

**Table 1:** Patients characteristics and perioperative status

	Case 1	Case 2
Age (Y)	63	84
Gender	male	female
BMI (Kg/m^2^)	22.8	19.0
Clinical diagnosis and stage	right renal tumor and spleen metastasis (cT1bN0M1)	concurrence of left renal tumor (cT2aN0M0) and ascending colon cancer (cT4aN2M0)
Operative procedure	right nephrectomy, splenectomy	left nephrectomy, right hemicolectomy
Incision of umbilicus	ZI	ZI
Total number of port	2	6
Placed the port status	GeIPOINT® and single additional 12mm port	conventional
Conversion of the surgical position intraoperatively	left to right lateral decubitus position	lithotomy position to lateral decubitus position
Drain	none	2
Operation time Nephrectomy/the other (min)	284 123/161	505 177/328
Total blood loss (ml)	91	898
Perioperative transfusion (unit)	none	2
Resume oral intake (day)	3	3
Hospitalization period (day)	7	16
Additional postoperative analgesic^a^	drip of 50 mg of flubiprofen × 1	none
Renal tumor	clear cell carcinoma (pT1aN0), spleen: cavernous hemangioma (M0)	clear cell carcinoma (pT1bN0), colon cancer: adenocarcinoma (pT3N0)

*BMI* body mass index, *ZI* zigzag incision

^a^Except for epidural anesthesia

Case 2: The next patient was an 84-year-old woman with concurrent left renal tumor and ascending colon cancer. She reported right flank pain and underwent screening CT. Enhanced CT showed a 75-mm-diameter left renal tumor and invasive focal ascending colon cancer (Fig. 1). The renal tumor was cT2aN0M0 RCC and the ascending colon cancer was cT4aN2M0. The patient’s BMI was 19.0 kg/m^2^ (Table 1).

### Surgical technique

**Fig. 2:**
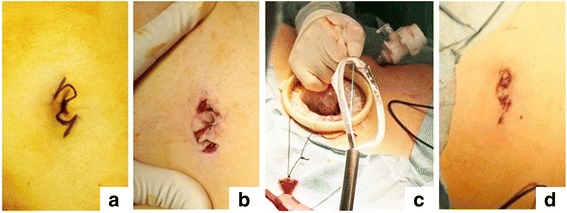
Perioperative view of the zigzag incision (ZI) created in Case 1: (**a**) pre-incision, (**b**) post-incision, (**c**) extraction of the right kidney from the ZI, and (**d**) post-suture

**Fig. 3:**
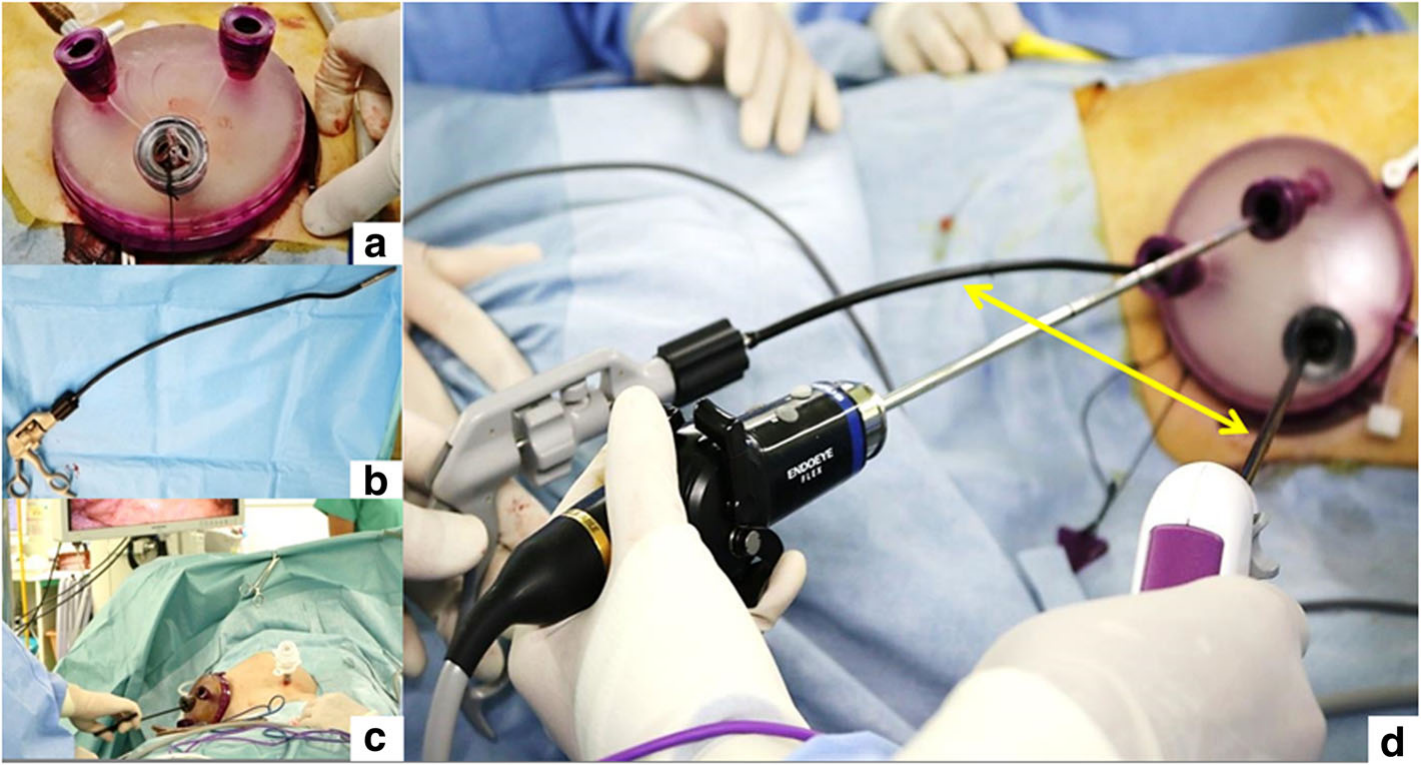
Surgical equipment used in Case 1. **a** GelPOINT, the multiport system, can be placed in three ports at most. **b** The ADACHI-TANKO Kanshi forms the bending of the shaft. **c** Whole ports image at splenectomy, GelPOINT and only one 12 mm assist port are placed. **d** Intraoperative photo of Case 1 using an ADACHI-TANKO Kanshi placed at GelPOINT shows that the distance between instruments can be maintained because of its form (double-headed allowed)

Case 1: We performed a radical right nephrectomy and splenectomy using the transumbilical approach with a ZI in a single-stage laparoscopic surgery (Fig. 2). A GelPOINT access platform (Applied Medical, CA, USA) was placed in the ZI, while a 12-mm assist port was placed at below the 12th rib costochondral margin on the left midclavicular line (Fig. 3a, c). We used ADACHI-TANKO Kanshi flexural forceps (ADACHI-INDUSTRY, Gifu, Japan) to reduce the interference between the laparoscope and the instruments (Fig. 3b, d). We first performed a right nephrectomy with the patient in the left lateral decubitus position and then converted from the left to the right position and performed a splenectomy. During the nephrectomy, beating of the renal artery was confirmed; thereafter, the inferior vena cava and renal vein were identified. One renal artery was blocked, but since the kidney did not shrink, another renal artery was identified and blocked. Soon thereafter, the kidney shrank and the renal vein was blocked and detached. Furthermore, at the time of the peritoneal incision and detachment of the upper pole of the kidney, the liver interfered and became difficult to maneuver around. Both specimens were extracted from the umbilical scar without extension of the wound (Fig. 2c). The surgery was completed without a blood transfusion or drain tube.

Case 2: We performed a right hemicolectomy followed by a left radical nephrectomy. The surgical position was converted from lithotomy for the hemicolectomy to lateral decubitus for the nephrectomy. The start of the midline incision included the umbilicus, and a port approximately 30 mm long accommodated the camera. Four other ports were used to perform the conventional laparoscopic hemicolectomy. An extra 12-mm port placed at below the 12th rib costochondral margin on the left anterior axillary line during the nephrectomy. First, an incision was made on a portion of the fused fascia, and then the space between the fascia and the Gerota fascia was carefully expanded during peeling to the renal pedicle. Upon reaching the renal pedicle, there was one renal artery and one vein and no obvious running abnormality. Moreover, the camera port was somewhat caudal compared to a regular port, but the usual percutaneous approach was used and the vessels were processed in nearly the same way, although some bleeding from the renal vein occurred during the peeling process. A few pieces of tissue sealing sheet (Tachosil®) were used to manage this. Because the resected specimen was too large to extract from the umbilical scar, the total length of the skin incision of the six ports was extended to 99 mm. Two drains were placed, one in the pelvic cavity and one in the renal cavity.

### Peri- and postoperative results

The perioperative results for both patients are shown in Table 1.

**Fig. 4:**
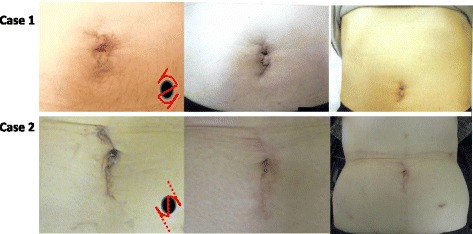
First (left) and sixth (center) month postoperative clinical images of Case 1 (above) and Case 2 (below). Moreover, the right figures are the whole abdominal images of both cases at sixth month postoperatively. The left lower figures are the incision images (the dotted line represents additional incisions) from the first month images

Case 1: The total operating time was 284 min: the right nephrectomy took 123 min, while the splenectomy took 161 min. Total blood loss was 91 mL. The pathological diagnosis of the renal tumor was clear cell carcinoma, pT1a, venous invasion (−). The splenic tumor was not diagnosed as metastatic RCC but rather as a cavernous hemangioma. The perioperative period was uneventful. Except for a general epidural, the only postoperative analgesic was a 50-mg flurbiprofen drip on postoperative day 2. He was discharged on postoperative day 7. Images of Case 1 show the condition of the umbilical region in the first postoperative month and the umbilical and whole abdominal regions in the sixth postoperative months, respectively (Fig. 4 above).

Case 2: The operating time totaled 505 min: the left nephrectomy took 177 min and the right hemicolectomy took 328 min. Total blood loss was 898 mL. The patient received 400 ml of red blood cell transfusion after surgery. The left RCC was diagnosed as clear cell carcinoma, pT1b, 65 × 65 mm, venous invasion (+). The pathological diagnosis of the ascending colon cancer was adenocarcinoma pT3 with no nodal involvement (0/48). The patient restarted oral intake on postoperative day 3. The only postoperative analgesic was the general epidural. The drain tubes were extracted on postoperative day 12 and the patient was discharged on postoperative day 16. Images of Case 2, as same as Case 1, show the condition of the umbilical region and whole abdominal regions (Fig. 4 below). Currently, both cases show no evidenced recurrence sites.

## Discussion

Here we performed single-stage laparoscopic surgery for several bilateral multifocal lesions. We approached the bilateral organ tumors by installing a GelPOINT in the navel at the center of the body’s surface. Hachisuka et al. reported that the ZI method is indicated for use in the umbilical region [5]. RPLS with a ZI was especially useful in Case 1 because it allowed the use of a shorter incision with the flexural forceps that allowed extraction of the spleen or lumen organs, such as part of the resected colon. However, a large organ, such as one > 70 mm affected by RCC, is difficult to extract from the original incision and would require an additional incision. We completed the operations safely in both cases using single-stage surgery. Nephrectomy required 123 min in Case 1 and 177 min in Case 2, which seem not so long compared to other reports [6]. Furthermore, the common incision for the bilateral lesions was only a skin incision and other surgical procedures do not shorten the total operating time because they require separate delamination and incision. Considering that it was necessary to change the patients’ positions for the bilateral tumors, the total operating time was acceptable. The perioperative courses in both case series were uneventful except for two units of blood transfused postoperatively in Case 2. When there is little perioperative bleeding, a drain tube may not be necessary. Experience with more cases and improvements in laparoscopic skills are necessary, but our proposed procedure may be a reasonable approach for the management of bilateral organ tumors. The shorter the total incision length, the less prolonged the postoperative ileus [7]. Walz et al. reported that single foramen surgery was superior to conventional multi-port surgery for adrenalectomies in terms of postoperative analgesic frequency and hospitalization period [8]. Our cases involved the complexities of multiple bilateral lesions. Nonetheless, in Case 2, which had the most ports (six), the total incision length was 99 mm, the same as that in conventional laparoscopic nephrectomy.

The ZI method was originally developed to make surgical wounds less noticeable. Figure 4 shows the condition of the umbilical region in the first and sixth postoperative months for this case series. An additional incision was required in Case 2 to enable extraction of the specimens (Fig. 4, below). Therefore, the umbilical scar of Case 2 was more obvious than that in Case 1. However, it can be confirmed that the scar of the additional incision site in Case 2 at 6 months postoperative is becoming less noticeable. The limitation of a ZI is that some cases require additional incisions. We could not make incisions along the circumference of the umbilicus; rather, they were made to both ends of the linear incisions. We designed the upper or lower half of the incision at the beginning of the surgery and then created the additional incision based on the requirement for specimen extraction. From a cosmetic standpoint, it is necessary to use a more refined ZI method that considers specimen size and skin striae direction. Furthermore, in order to prove the superiority of these procedures, a larger clinical trial using a quantitative evaluation method such as Derriford Appearance Scale (DAS 59) should be necessary [9].

## Conclusions

This is the first report describing single-stage surgery using a ZI and a transumbilical approach that can be accomplished safely and with a better aesthetic outcome than previous surgical methods for bilateral organ lesions. In conclusion, based on our experiences, this novel procedure could be considered for patients with bilateral lesions. However, in order to prove superiority of these procedures, this should be tested in a clinical trial setting.

## Abbreviations

BMI: Body mass index
CT: Computed tomography
RCC: Renal cell carcinoma
RPLS: Reduced port laparoscopic surgery
US: Ultrasonography
ZI: Zigzag incision
